# Protocol for a randomised controlled feasibility trial of parent-delivered early language intervention for children with Down syndrome (PACT-DS)

**DOI:** 10.1186/s40814-023-01419-7

**Published:** 2023-12-07

**Authors:** Kelly Burgoyne, Emma Pagnamenta, Kirstie Hartwell, Vesna Stojanovik

**Affiliations:** 1 Manchester Institute of Education, University of Manchester, Manchester, M13 9PL UK; 2 University of Reading, Reading, UK

**Keywords:** Down syndrome, Intervention, Parents, Language, Literacy

## Abstract

**Background:**

Down syndrome is the most common genetic cause of intellectual disability, affecting 700–800 babies annually in the UK (Wu J, Morris J, Eur J Hum Genet 21:1016–9, 2013). Children with Down syndrome have difficulties developing language skills. These difficulties can have long-term negative consequences for all aspects of their lives including social development, education and employment opportunities, and emotional wellbeing and mental health (Irwin LG et. al 2007). These aspects all have the potential to be improved through targeted early language intervention. Parents and Children Together (PACT) is a parent-delivered early language teaching programme for typically developing pre-school children at risk of language delays. A previous project (Burgoyne K, J Child Psychol Psychiatry 59(5):545–55, 2018) showed that PACT leads to improvements in children’s language and early literacy skills. Our team has worked closely with six families to adapt PACT for children with Down syndrome. The aim of the current study is to conduct a feasibility randomised controlled trial (RCT) to evaluate the feasibility of a definitive RCT and explore initial evidence of the adapted programme’s potential to support children’s language and literacy development.

**Method:**

This is a two-arm feasibility randomised controlled trial in which children will be randomly allocated to either a PACT-DS group or to a waiting control group (who will receive intervention at the end of the project). We aim to recruit 28–30 children with Down syndrome (aged 3–6 years) and their parents/caregivers to take part. The PACT-DS group will be trained to deliver PACT-DS every day (20 min a day; 5 days a week) to their child over 30 weeks. We will collect data using assessments of child language and early literacy, measures of parent wellbeing, qualitative surveys and interviews, and monitoring data to explore trial feasibility (including recruitment and retention of families and adherence and acceptability of intervention) and cost and benefits. Data will be collected before intervention, immediately after the 30-week intervention programme, and 6 months after intervention ends. Clear progression criteria will be used to assess suitability for a definitive trial.

**Discussion:**

This study represents initial steps in developing a definitive trial of the PACT-DS programme and will add to the limited evidence base on early language intervention for children with Down syndrome. This programme of research has the potential to make significant advancements in early language intervention research and practice for this group.

**Trial registration:**

The trial is registered with the ISRCTN registry: study ID ISRCTN63251282. Registered on 14 July 2023.

**Supplementary Information:**

The online version contains supplementary material available at 10.1186/s40814-023-01419-7.

## Background and rationale

Down syndrome is the most common genetic cause of intellectual disability, affecting 700–800 babies annually in the UK [1]. All individuals with Down syndrome develop more slowly than in typical development, but there are considerable individual differences. Further, not all aspects of development are equally delayed; instead, there is a pattern of strengths and weaknesses, with social understanding and visual processing often regarded as relative strengths and speech and language development frequently considered the greatest challenge (e.g. [2]). Language development largely follows the typical course but is delayed from the outset [3].

Language difficulties affect all aspects of life, including social opportunities, learning and cognitive development, mental health, and independence and inclusion in the community (e.g. [4, 5]); these aspects all have the potential to be improved through targeted language intervention. In line with this, current UK policy highlights the importance of early intervention [6] and calls for action to improve language acquisition and reading skills in the early years, including supporting parents to develop their children’s language skills at home [7]. This has particular relevance to children with Down syndrome who have significant language learning difficulties and need support for language development from an early age to reach their full potential. There is, however, a lack of evidence-based language intervention for children with Down syndrome, and whilst parents are well placed to support early language development [8], they need support to do so.

Finding ways to support language development from an early age is critical for children with Down syndrome. Early childhood is characterised by rapid brain growth and heightened neuroplasticity [9], making this a particularly fruitful time for language learning. Further, some of the language and memory difficulties seen in Down syndrome are not present in the early years but emerge downstream and could potentially be ameliorated through early intervention [10, 11]. However, very little evidence-based support for early language development is currently available (e.g. [12, 13]). A recent systematic review of language interventions for children with Down syndrome found only eight studies meeting quality criteria [13]. Findings from this review demonstrate that children with Down syndrome can make significant gains in language from targeted intervention; however, the youngest children in these studies were 5 years old, and interventions were largely delivered in school. Involving parents in early intervention is a more cost-effective approach than practitioner delivery [14] and is especially important in the current climate where face-to-face interventions and therapy are restricted due to overstretched services. Further, the earlier an intervention happens, the better the return on investment [15]. High-quality studies evaluating parent-delivered early language intervention for young children with Down syndrome are therefore urgently needed.

This paper outlines the protocol for a feasibility study of the PACT-DS programme: a 30-week early language intervention which is designed for parents/caregivers to deliver to their young child at home. The programme was originally developed for typically developing pre-school children at risk of language delays. A randomised controlled trial (RCT) with 208 socially disadvantaged pre-school children and their parents/caregivers [16] showed effects of the programme on children’s language (Cohen’s *d* (effect size) = 0.21) and narrative skills (*d* = 0.36) immediately following intervention. Effects on language outcomes were maintained 6 months after the programme ended (*d* = 0.36), and at this point, the intervention group also scored higher on measures of early literacy (*d*’s = 0.35 and 0.42).

### Parent co-creation

Between September 2022 and June 2023, we worked closely with a small group of parents with a 4–6-year-old child with Down syndrome (*N* = 6 children) to pilot the PACT programme and co-produce a modified version of the programme which is suitable and acceptable to parents and children with Down syndrome (which we call PACT-DS). Parents provided detailed information and feedback on their experiences and adaptations to the programme and the materials that will be used in the trial. This included the following:

- Delivering 5 weeks of the unadapted original PACT programme and a further 5 weeks of adapted programme materials and providing detailed data to evaluate aspects working well and areas for adaptation.
- Participating in two focus groups and a workshop to discuss the programme and proposed adaptations and the protocol for the feasibility randomised controlled trial.
- Reviewing the resources and teaching targets (books and words) in the original PACT 30-week programme and indicating their thoughts on suitability for children with Down syndrome and whether to keep or replace them in the adapted programme.
- Developing video resources to support families taking part in the feasibility trial to deliver the programme, e.g. videos of parents delivering the programme with their child, and tips for delivery.
- Copresenting with the research team to share their experiences of delivering the programme at the Down Syndrome Research Forum [17].

## Aims and objectives

The aim of the current study is to establish the feasibility of a definitive RCT of PACT-DS. To meet this aim, the study has the following key objectives:

1. To deliver a two-armed feasibility RCT of the adapted PACT programme (PACT-DS), including 28–30 children with Down syndrome and their families
2. To determine recruitment, retention, and adherence rates and acceptability of the intervention and trial procedures for families
3. To determine the suitability of assessment measures and explore potential benefits and costs of the programme and establish the sample size needed for a definitive trial

## Trial design

**Fig. 1:**
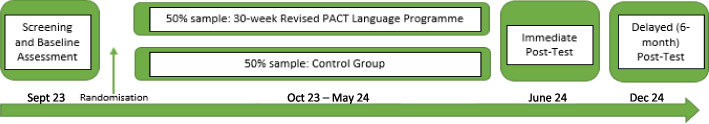
Feasibility RCT design

This study is a two-armed feasibility RCT in which children and their parents will be randomly allocated to PACT-DS or to a waiting control group. Parents in the PACT-DS group will be trained to deliver the programme over 30 weeks. The protocol has been developed in line with the Standard Protocol Items: Recommendations for Interventional Trials (SPIRIT) 2013 checklist (see Supplementary materials) and in collaboration with families taking part in piloting and adapting the intervention programme and the project steering group. See Fig. 1 below for a flow chart of the study.

## Setting

We will recruit families across two sites with a relatively large geographical spread (North-West: Greater Manchester/Lancashire; South-East: Berkshire/Greater London/Oxfordshire/Hampshire) to ensure access to enough participants. The study is being carried out at the University of Manchester and the University of Reading, UK.

## Participants

### Sample size

The literature recommends a minimum of 24 participants [18–20] in order to derive a standard deviation. We aim to recruit 28–30 families with a 3–6-year-old child with Down syndrome to take part, in order to account for potential attrition. Families will be recruited by advertising the project through local parent support groups, speech and language therapy organisations (e.g. via the Down Syndrome Clinical Excellence Network, Speech and Language Therapy service managers, Royal College of Speech and Language Therapists), and national Down syndrome organisations (e.g. Down syndrome Education International, LETS Go! UK).

### Eligibility criteria

#### Inclusion criteria

1. Child has a diagnosis of Down syndrome.
2. Child aged 3–6 years at the start of the study
3. Family home postcode is within 40 miles of the University of Manchester/University of Reading.
4. Parents can read and speak English.
5. Child has a minimum expressive vocabulary of 10 (English) words/signs (measured via parent-completed Reading Communicative Development Inventory; [21]).
6. Child has a minimum cognitive level of 18 months (as assessed by the research team using the Mullen Scale of Early Learning; [22]). As a broad indication, the types of skills this would reflect would be interest in books, being able to attend to a picture and able to use two hands together to, for example, grasp an object.

#### Exclusion criteria

1. Twins will be excluded as they had higher levels of attrition in a previous trial [16].

### Intervention

Each intervention session follows a consistent structure and routine (see Table 1). In ‘Reading Together’, parents and children read storybooks together and talk about the story. Parents are provided with strategies and guidance to support their child’s active involvement and promote conversation around the story. Words that feature in or are related to the storybooks are targeted for direct teaching in the ‘Words’ component of the programme. These new words are taught using simple repetitive games and activities and picture resources to reinforce understanding and practice saying new words. The ‘Using Word’s’ component provides structured activities and visual resources to foster understanding of story structure and provide practice creating sentences that summarise key story elements. Parents work through the manualised PACT-DS programme with their child at home every day (5 days a week) for 20 min over 30 weeks. The programme is organised in 6 × 5-week ‘blocks’: in each block, weeks 1–4 introduce new learning, and week 5 focuses on revision and consolidation.

**Table 1:** Overview of a PACT-DS session

**Reading Together**	Read the book together and talk about the story
Words	Play games with new words
Using Words	Create sentences together to sequence and retell stories

The PACT-DS programme incorporates several features that support learning and development in children with Down syndrome: (1) PACT activities are highly social and interactive which individuals with Down syndrome typically find motivating [23]; (2) it supplements naturalistic (shared reading) activities with structured and explicit teaching, which is necessary to improve language outcomes for this group [24]; (3) the programme is highly visual, playing to relative strengths in visual-spatial memory [25]; (4) there are frequent opportunities for repetition and consolidation [26]; (5) the programme can be tailored to individual strengths and weaknesses accounting for the wide variability seen in Down syndrome; (6) intervention sessions consist of several short activities, supporting attention and behaviour; and (7) intervention is intensive (daily) and sustained (150 intervention sessions over 30 weeks).

### Comparator

The comparator is a waiting-list control group who will be offered three training sessions on language development during the 30-week intervention period, and at the end of the trial will be offered a choice of PACT-DS or an alternative evidence-based intervention programme designed to support reading and language development in school-aged children with Down syndrome; [27]).

### Procedure

**Fig. 2:**
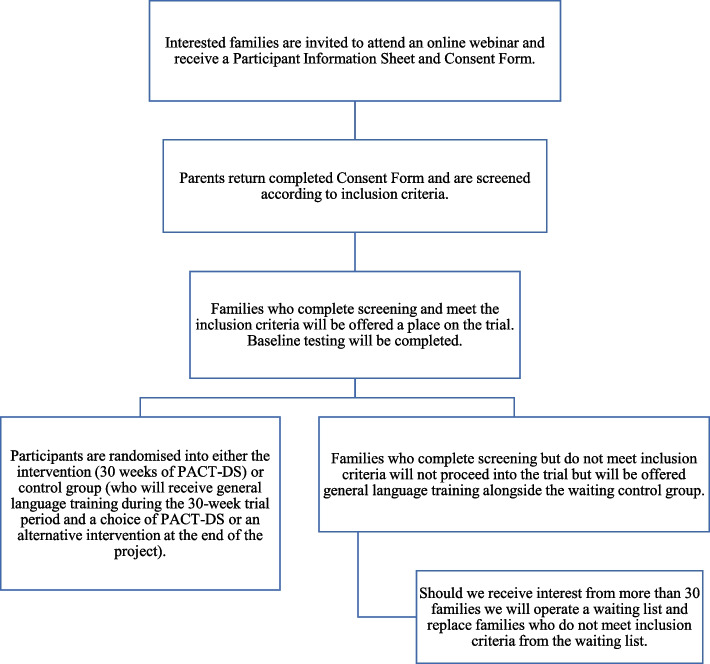
Recruitment and screening procedure

The recruitment and screening procedure are summarised below in Fig. 2. We will deliver online webinars for interested families, where we will outline the process of the study and explain what will happen if they are involved (including key issues such as randomisation and assessments). If families wish to take part, we will ask them to return a signed consent form which will screen participants according to initial inclusion criteria (criteria 1–4 above) including their geographical location (postcode) and age of child. Those who meet criteria will then complete the remaining screening procedures (criteria 5–6 above). Families who meet screening criteria will then be accepted into the trial and complete baseline assessments. Families who complete screening but do not meet inclusion criteria will not proceed into the trial but will be offered general language training alongside the waiting control group. Should we receive interest from more than 30 families, we will operate a waiting list and replace families who do not meet inclusion criteria from the waiting list.

### Randomisation

Following baseline assessments, randomisation will be at the child level, stratified by site (University of Manchester/University of Reading) and the child’s main form of communication (sign/spoken language/sign + spoken language) as reported by parents. Randomisation will be conducted by an independent randomisation service provider (e.g. sortition; a secure web-based clinical trial randomisation software developed by the University of Oxford). Children and their parents will be randomly allocated to the PACT-DS programme group or to the waiting-list control group. Following randomisation, the family will be contacted by the research team to explain the group allocation and next steps. Regardless of group allocation, all children and families will be reassured they should continue with any additional therapy or interventions they are receiving during the trial (e.g. speech and language therapy).

#### Intervention group

Families allocated to the experimental group will be trained by the research team to deliver PACT-DS. Training involves an in-person group training session, delivered to parents/caregivers in small groups. Any adult family member who will be involved in delivering the programme to the child (e.g. older adult sibling, grandparent) will be asked to attend training; there is no limit on the maximum number of family members that can attend training and deliver the programme per child.

The programme is organised into 6 × 5-week blocks. Parents will receive materials for block 1 of the programme at training; subsequent blocks will be sent to parents as they progress through the programme to support continued engagement and avoid overwhelming them. Parents will be asked to work through the programme with their child at home, delivering daily (5 × week) sessions of 20–25 min, over 30 weeks of teaching. All materials and resources needed to deliver the programme will be provided. Parents will be asked to complete a daily record form to record details of delivery (including whether the session was completed and whether the child enjoyed it). The research team will offer ongoing telephone and email support and will conduct a minimum of two home visits with each family during the delivery phase to observe intervention delivery and provide individualised feedback and support. The protocol for home visits will be standardised so that researchers carry out each visit and provide feedback uniformly.

#### Control group

Families in the waiting control group will be offered general training on supporting language development for children with Down syndrome. This will involve three 1-h sessions, delivered online via Zoom call to parents as a group, over the delivery phase. In addition, on completion of the trial, they will have the option of receiving PACT-DS or an evidence-based reading and language intervention for children with Down syndrome [27]. In this way, all families will receive some form of support from the outset. We will also offer all families a written report of their child’s assessment data at each assessment point.

#### Adherence to the intervention and contamination

Adherence to intervention will be assessed using measures of frequency and number of intervention sessions completed, programme completion rate, programme dropout (with reasons), and extent to which parents delivered intervention as intended. This data will be collected through daily record forms, records of email and telephone support, and observations of intervention delivery conducted during home visits.

The risk of contamination is deemed very low as only families in the programme group will have access to programme training and intervention materials (some of which are not reusable). We will also stress the importance of maintaining group allocation, and not sharing intervention resources, at recruitment and training. We will evaluate potential contamination by asking families (via a usual practice survey) at study entry whether they have been exposed to information about PACT-DS and/or programme materials and resources. We will repeat these questions at posttest for families in the control group.

### Data collection

One of the goals of the feasibility study is to evaluate the most suitable measures for capturing intervention outcomes. A range of measures including child assessments, parent-completed measures, and measures of parent wellbeing will be evaluated to determine which are most suitable for determining primary and secondary outcomes.

**Fig. 3:**
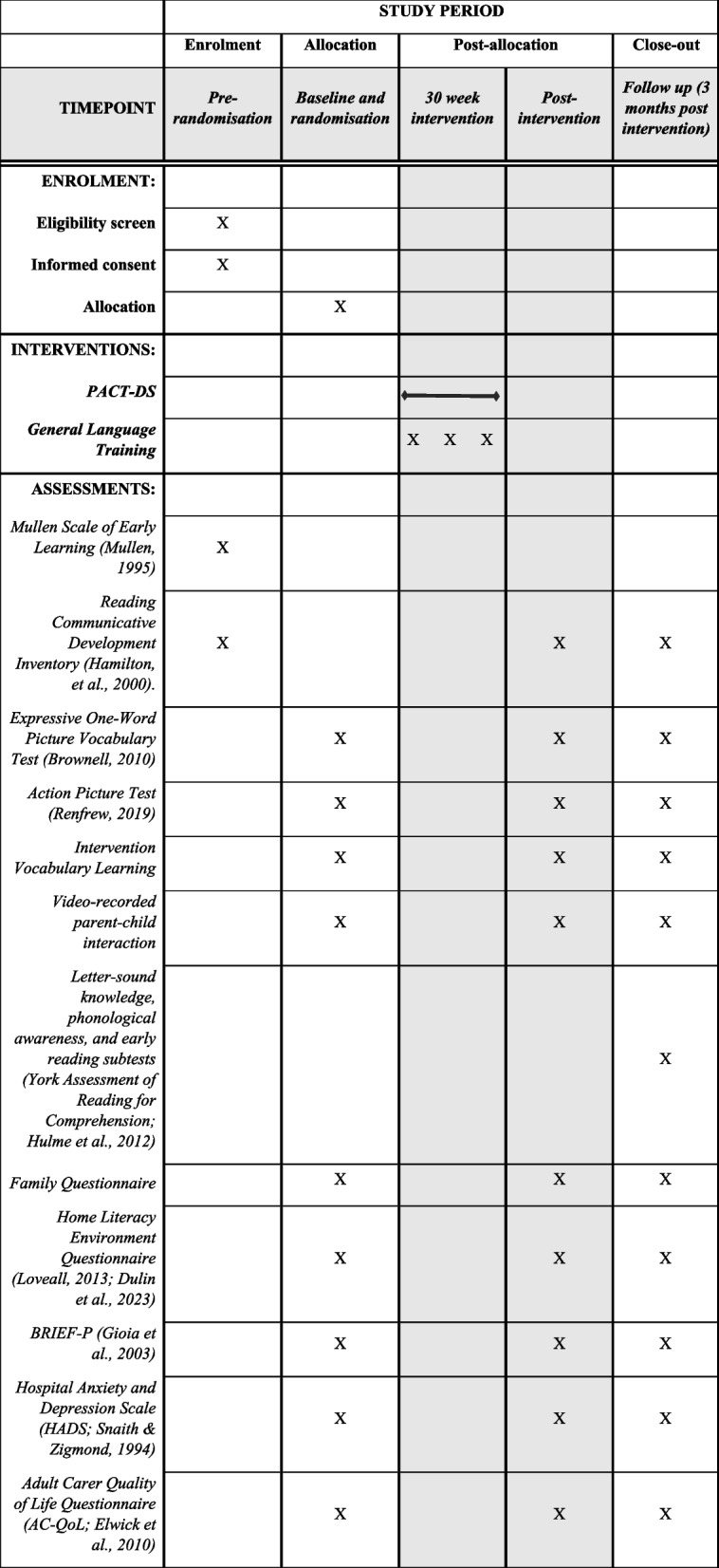
Schedule of enrolment, interventions, and assessments

All children in the study will be assessed at baseline, immediately after the 30-week intervention, and 6 months after the intervention ends using the following measures (see Fig. 3 and the SPIRIT checklist in supplementary materials):

- *Expressive One-Word Picture Vocabulary Test* [28] will be used to measure expressive word knowledge. This picture-based test assesses single word naming of objects, actions, and concepts.
- *Action Picture Test* [29] will provide information about the child’s spoken language (information score) and grammatical skills (grammar score). The child is asked to describe ten picture cards of everyday scenarios by answering a question about each one, for example ‘What is the girl doing?’.
- A bespoke assessment of intervention vocabulary learning will examine expressive and receptive learning of a selection of words taught in the intervention programme.
- Video-recorded parent–child interactions during 10 min of (a) shared reading and (b) free play will provide natural language samples which will be transcribed and coded (using CLAN software) for measures of child language (including no. of words, no. of different words, mean length of utterance, and spontaneous and imitated signs) and parent measures including responsiveness and use of language boosting strategies (e.g. expansion).
- At 6-month delayed posttest, children will also complete measures of early literacy (letter-sound knowledge, phonological awareness, and early reading subtests from the York Assessment of Reading for Comprehension; 30) to explore potential effects of PACT-DS on emergent literacy skills.

Testing at each time point will take place in the university or at the child’s school or home. All tests will be administered by trained research staff; at immediate and delayed posttest, researchers blind to group allocation will collect assessment data. All assessments will be video recorded for quality assurance purposes.

At baseline, immediate, and delayed posttest, parents will be asked to complete a number of additional measures as follows:

- The Reading Communicative Development Inventory [21] is a parent-report measure of spoken and signed vocabulary (understanding and use). Any words targeted by the intervention programme that are not present will be added to the inventory in order to also measure intervention vocabulary in both the intervention and control groups. Inclusion of intervention vocabulary within the Communicative Development Inventory will mitigate for parents of children in the control group becoming explicitly aware of these words.
- A family questionnaire to measure demographic information (e.g. indices of socio-economic status including parental education, number of children in family, etc.) and child health (e.g. vision and hearing, lengthy hospitalisations, medication). This questionnaire will also gather information on nursery/school placement, access to intervention and therapy (e.g. speech and language therapy, portage), and, if in receipt of speech and language therapy, frequency of input and targets. Questions relating to parent knowledge, skills, and confidence in relation to language development and supporting their child’s language development, based on the Theoretical Domains Framework [30], will also be included.
- A Home Learning Environment questionnaire, adapted from previous research [31, 32] to measure the home learning environment (e.g. frequency of shared reading, parent use of shared book reading activities).
- BRIEF-P [33] to examine child executive functioning. Parents rate their child’s executive functions (for example inhibition, emotional control, working memory, planning) in the context of their everyday environments.
- The Hospital Anxiety and Depression Scale (HADS; 35) to measure parent anxiety and depression and the Adult Carer Quality of Life Questionnaire (AC-QoL; 36) to measure parental quality of life. These measures are included as variables which may potentially affect intervention delivery and/or change as a result of delivering intervention and would inform planning for cost-effectiveness analysis within a future definitive RCT.

The intervention group will also be asked to complete a questionnaire based on the Theoretical Domains Framework [30] to measure parental knowledge, skills, social identity, beliefs about capabilities and consequences, memory attention and decision processes, environmental context and resources, emotion, behavioural regulation, intention, and goals related to delivering the PACT-DS intervention immediately after intervention training, 10 weeks into intervention delivery, and at immediate posttest.

#### Qualitative evaluation

Qualitative work with parents will explore perceptions of the programme and the acceptability and feasibility of trial procedures. Post-intervention, we will use simple online surveys to ask parents about their experiences of taking part, including recruitment and randomisation processes, training and intervention delivery, and completion of outcome measures. Acceptability of the intervention will be measured using the theoretical framework of acceptability questionnaire [34]. Records of email and telephone support will also inform our understanding.

In addition, between immediate and delayed posttest, we will invite a purposive sample of parents (*N* = 8; 4 × PACT-DS group; 4 × waiting control) who are representative of the range of diversity in the sample to participate in a 1:1 semi-structured interview (face to face or by telephone/video call) to follow up on key themes emerging from the parent intervention evaluation surveys, including acceptability, parental knowledge, skills, and identity as implementers of intervention.

### Outcomes

Feasibility objectives and acceptability of intervention outcome measures will be assessed using descriptive analysis, focusing on confidence interval estimation rather than formal hypothesis testing to explore initial evidence of the potential effects of the programme. The feasibility study’s outcomes will determine whether it would be viable to progress to a definitive trial and whether/what adjustments may need to be made to the procedures, materials, data collection methods, intervention delivery, intensity, and parent training. Descriptive statistics will also be calculated for possible future sample size estimation.

#### Primary outcome measures

The feasibility of a future definitive trial of the PACT-DS programme is assessed using the following:

- 1. The number of participants interested in taking part, consented, completed screening, eligible, and randomised
- 2. Adherence to and acceptability of the PACT-DS intervention via measures of attendance at training, number of intervention sessions completed, number of participants withdrawing from intervention, and intervention fidelity as well as records of email and telephone support provided to families, surveys, and qualitative interviews.
- 3. The acceptability and experience of the trial process to participants, including randomisation and completion of outcome measures
- 4. The optimal primary outcome measure in a future trial, determined by assessing the performance of selected candidate primary outcome measures with respect to the level of acceptability to participants (completion rates, perceived burden) and reliability.
- 5. Estimated sample size for a future trial by measuring participant withdrawal, data completeness at follow-up (participant attrition), and group differences and 95% confidence intervals for child language measures at immediate and delayed follow-up.
- 6. The potential benefits and costs in a future trial including benefits for child and parent outcomes and costs of intervention including intervention materials and resources and staff time for training and support (in line with recent cost guidance from the Education Endowment Foundation).

Progression to definitive trial will be informed by the results of three main progression criteria:

- Recruitment: At least 50% of the proposed sample (*n* = 28–30) recruited to (i.e. take up an offered place in) feasibility study = green, 40–49% = amber, and below 40% = red
- Intervention adherence: Will be based on the experimental group delivering the programme, i.e. 50% + of the intervention group complete =  > 17 weeks of the programme (the average completion rate in Burgoyne et al., 2018) = green, 40–49% = amber, and below 40% = red
- Retention will be based on the proportion of the recruited sample that completes delayed post-testing (i.e. children with quantitative data at delayed post-test), i.e. 80% +  = green, 50–79% = amber, and < 50% = red.

#### Secondary outcome measures

The outcome measures that will be evaluated within this feasibility study are listed below. The following measures are all collected at baseline (prior to randomisation) and at immediate posttest (immediately following the 30-week intervention phase) and follow-up posttest (6 months after the immediate posttest) with all participants (in both groups) with the exception of the child emergent literacy measure (assessed at 6-month follow-up only):

- Child language is as follows:
  1. Child vocabulary, assessed using the parent-completed Reading Communicative Development Inventory [21]
  2. Child expressive vocabulary, assessed using the *Expressive One Word Picture Vocabulary Test* [28]
  3. Child expressive information and grammar, assessed using the *Action Picture Test* [29]
  4. Child intervention vocabulary knowledge, assessed using a researcher-developed task
  5. Parent–child interaction, providing measures of child language and parent measures (e.g. responsiveness, use of language boosting strategies), assessed using video recordings of parents and children sharing books and playing together
- Child emergent literacy (assessed only at 6-month follow-up) is as follows:
  6. Child emergent literacy, assessed using the York Assessment of Reading for Comprehension (assessed at 6-month follow-up only) [35]
- Home learning environment is as follows:
  7. Home learning environment, assessed using a Home Learning Environment questionnaire [31, 32]
- Child executive functioning is as follows:
  8. Child executive functioning, assessed using the Behaviour Rating Inventory of Executive Functioning-Preschool [33]
- Parent measures are as follows:
  9. Parent knowledge, skills, and confidence in relation to language development, as assessed through a questionnaire based on the Theoretical Domains Framework [30]
  10. Parent anxiety and depression, as measured by the Hospital Anxiety and Depression Scale (HADS) [36]
  11. Parental quality of life as assessed by the Adult Carer Quality-of-Life Questionnaire (ACQoL) [37]

### Data analysis and presentation

CONSORT reporting guidelines will be used to report outcomes from the trial. Feasibility objectives and acceptability of intervention outcome measures will be assessed using descriptive analysis, focusing on confidence interval estimation rather than formal hypothesis testing. Qualitative data from interviews will be transcribed and coded for key themes related to participants’ experiences of the intervention and the trial.

## Data management and security

### Data transfer, storage, and archiving

The project will produce both qualitative and quantitative data. The University of Manchester and the University of Reading have a data sharing agreement. Where participants consent to data sharing, data will be transferred using secure university-approved data sharing services.

All data collected will be pseudo-anonymised as soon as possible. The data and metadata will be stored electronically on secure university networks in password-protected files. The PC/laptops used will also be password protected. Files containing personal data will be password protected and stored separately from pseudo-anonymised project data. A single linking file (password protected) will be stored separately. Hard copies of data (e.g. participant record forms) will be stored securely in a locked filing cabinet in a secure university building; these will be scanned to create electronic versions as soon as possible after which hard copies will be destroyed.

Data quality will be monitored routinely. Video recordings will be uploaded to secure storage and then will be deleted from recording equipment. Recordings will be destroyed after transcription. Transcripts will be anonymised before analysis. Participant details will be pseudo-anonymised until the analyses have been completed. The linking file will be destroyed at the end of the trial, and only anonymized data will be kept. The anonymized final data set will be stored indefinitely in a repository.

The PI (Dr. Burgoyne) will have overall responsibility for data management at the University of Manchester and for data archiving. Prof Stojanovik will be responsible for data management at the University of Reading and will assist with preservation and preparation for archiving purposes.

### Study governance

The project management team consists of the principal investigator (PI), co-investigators (Co-Is), post-doctoral research assistant (P. D. R. A.), and research assistant (R. A.). The PI will meet with the PDRA and RA weekly, and the whole team will meet monthly to discuss the progress and management of the project. The project has been reviewed by research ethics committees at the University of Manchester and the University of Reading to ensure it meets ethical approval.

We have also established a project advisory group of nine experts who have a variety of expertise and interests relevant to the project, to maximise the benefits of the project and support knowledge transfer. Members include speech and language therapists, educators, academics, local support group leads, and parents of children with Down syndrome. The project management team will meet with the advisory group every 6 months during the course of the project to discuss progress of the project and seek advice and guidance around key methodological issues and project milestones and outputs and dissemination.

## Dissemination

We have a broad and comprehensive dissemination strategy from the outset that aims to share the study and its findings with the Down syndrome community as well as with academic and professional audiences. We have a study website (https://sites.manchester.ac.uk/pct/pact-ds/) and social media account (@PACTLanguage) to support engagement and dissemination. We will produce project newsletters and at least two online webinars to (a) explain the study and promote recruitment, (b) provide regular updates on project milestones, and (c) share the outcomes of the study. These will be shared with Down syndrome support groups across the UK and advertised on social media and via Down syndrome and speech and language organisations. The results of the study will be published in peer-reviewed scientific journals, practitioner publications, conference presentations, and participation in Down syndrome clinical excellence network meetings. We will also develop an online workshop for the academic research community to share critical methodological learning and resources arising from the project about working with parents to adapt an existing intervention.

## Discussion

This feasibility study will significantly advance our understanding of ways to enable parents of young children with Down syndrome to support their child’s language development at home. It is the next step in a programme of work to cocreate and test a parent-delivered language intervention that has been adapted specifically for young children with Down syndrome, thus addressing the need for evidence-based interventions to enhance communication outcomes for this population. The mixed-methods approach will allow us to explore parental perceptions, experience, and acceptability of the intervention, in addition to theoretically informed measures of behavioural change in relation to the role of parents as implementers of the intervention. The study will provide critical knowledge that will form the foundation of a definitive RCT of the intervention programme. If rates of recruitment, adherence, and retention are adequate, we will seek funding to conduct a definitive trial. The findings of this feasibility trial will be of relevance to policy-makers, practitioners, and researchers interested in interventions to enhance language and communication outcomes, as well as to families and the Down syndrome community.

## Trial status

This paper refers to protocol version 1 dated 14 July 2023. Recruitment will begin in July 2023 with completion expected by the end of September 2023. Post-intervention assessments are expected to be completed by the end of June 2024 and delayed post-intervention assessments by the end of December 2024.

### Supplementary Information

**Additional file 1.** 

## Data Availability

Not applicable.

## References

[1] Wu J, Morris J (2013). The population prevalence of Down’s syndrome in England and Wales in 2011. Eur J Hum Genet.

[2] Chapman RS, Hesketh LJ (2000). Behavioural phenotype of individuals with Down syndrome. Ment Retard Dev Disabil Res Rev.

[3] Abbeduto L, Warren SF, Conners FA (2007). Language development in Down syndrome: from the prelinguistic period to the acquisition of literacy. Ment Retard Dev Disabil Res Rev.

[4] Law J, Rush R, Schoon I, Parsons S (2009). Modeling developmental language difficulties from school entry into adulthood: literacy, mental health, and employment outcomes. J Speech Lang Hear Res.

[5] Irwin LG, Siddiqi A, Hertzman C (2007). Early child development: a powerful equalizer. Final report for the WHO’s Commission on Social Determinants of Health. World Health Organization, Commission on Social Determinants of Health.

[6] House of Commons Library (2019). Early intervention. Briefing Paper (7647).

[7] Department of Health and Social Care. Prevention is better than cure: our vision to help you live well for longer. Policy Paper; 2018. https://assets.publishing.service.gov.uk/media/5be00437e5274a6e174bdac1/Prevention_is_better_than_cure_5-11.pdf.

[8] Roberts MY, Curtis PR, Sone BJ, Hampton LH (2019). Association of parent training with child language development: a systematic review and meta-analysis. JAMA Pediatr.

[9] Brown TT, Jernigan TL (2012). Brain development during the preschool years. Neuropsychol Rev.

[10] Mason-Apps E, Stojanovik V, Houston-Price C, Buckley S (2018). Longitudinal predictors of early language in infants with Down syndrome: a preliminary study. Res Dev Disabil.

[11] Roberts LV, Richmond JL (2015). Preschoolers with Down syndrome do not yet show the learning and memory impairments seen in adults with Down syndrome. Dev Sci.

[12] O’Toole C, Lee ASY, Gibbon FE, van Bystervedlt AK, Hart NJ (2018). Parent mediated interventions to promote communication and language development in children with down syndrome aged between birth and six years'. Cochrane Database Syst Rev.

[13] Smith E, Hokstad S, Næss KAB (2020). Children with Down syndrome can benefit from language interventions: results from a systematic review and meta-analysis. J Commun Disord.

[14] Gibbard D, Coglan L, MacDonald J (2004). Cost-effectiveness analysis of current practice and parent intervention for children under 3 years presenting with expressive language delay. Int J Lang Commun Disord.

[15] Heckman JJ (2008). Schools, skills, and synapses. Econ Inq.

[16] Burgoyne K, Gardner R, Whiteley H, Snowling M, Hulme C (2018). Evaluation of a parent-delivered early language enrichment programme: evidence from a randomised controlled trial. J Child Psychol Psychiatry.

[17] Hartwell K, Livesey L, Pagnamenta E, Stojanovik V, Burgoyne K. Parent-delivered early language intervention for children with Down syndrome (PACT-DS): a pilot study. [Online presentation]. Down Syndrome Research Forum, Online, United Kingdom. 2023.https://www.down-syndrome.org/en-gb/research/forum/2023/. 10.1186/s40814-023-01419-7 PMC10702108 38062458

[18] Lancaster GA, Dodd S, Williamson PR (2004). Design and analysis of pilot studies: recommendations for good practice. J Eval Clin Pract.

[19] Sim J, Lewis M (2012). The size of a pilot study for a clinical trial should be calculated in relation to considerations of precision and efficiency. J Clin Epidemiol.

[20] Julious SA (2005). Sample size of 12 per group rule of thumb for a pilot study. Pharm Stat.

[21] Hamilton A, Plunkett K, Schafer G (2000). Infant vocabulary development assessed with a British communicative development inventory. J Child Lang.

[22] Mullen EM (1995). Mullen Scale of Early Learning.

[23] Fidler DJ (2006). The emergence of a syndrome-specific personality profile in young children with Down syndrome. Downs Syndr Res Pract.

[24] Buckley S (2008). The power of behavioural approaches – we need a revival. Down Syndr Res Pract.

[25] Jarrold C, Baddeley A, Phillips C (1999). Down syndrome and the phonological loop: the evidence for, and importance of, a specific verbal short-term memory deficit. Down Syndr Res Pract.

[26] Chapman RS, Sindberg H, Bridge C, Gigstead K, Hesketh L (2006). Effect of memory support and elicited production on fast mapping of new words by adolescents with Down syndrome. J Speech Lang Hear Res.

[27] Burgoyne K, Duff F, Clarke P, Smith G, Buckley S, Snowling M, Hulme C. A Reading and Language Intervention for Children with Down Syndrome. Down Syndrome Education International. 2012. https://www.down-syndrome.org/en-gb/resources/teaching/rli/. 10.1111/j.1469-7610.2012.02557.x PMC3470928 22533801

[28] Brownell R (2010). Expressive one-word picture vocabulary test-4.

[29] Renfrew C (2019). Action picture test.

[30] Huijg JM, Gebhardt WA, Crone MR, Dusseldorp E, Presseau J (2014). Discriminant content validity of a theoretical domains framework questionnaire for use in implementation research. Implement Sci.

[31] Loveall SJ. Reading skills in Down syndrome: an examination of orthographic knowledge. Doctoral dissertation, University of Alabama. Tuscaloosa, Alabama: ProQuest Dissertations & Theses Global; 2013.

[32] Dulin M, Loveall SJ, Mattie LJ (2023). Home-literacy environments and language development in toddlers with Down syndrome. Front Psychol.

[33] Gioia GA, Espy KA, Isquith PK (2003). Behaviour rating inventory of executive function-preschool version.

[34] Sekhon (2022). Development of a theory-informed questionnaire to assess the acceptability of healthcare interventions. BMC Health Serv Res.

[35] Hulme C, Stothard SE, Clarke P, Bowyer-Crane C, Harrington A, Truelove E, Snowling MJ (2009). YARC York assessment of reading for comprehension: Early Reading.

[36] Snaith RP, Zigmond AS (1994). The Hospital Anxiety and Depression Scale.

[37] Elwick H, Joseph S, Becker S, Becker F (2010). Adult Quality of Life Questionnaire. The Princess Royal Trust for Carers in association with the School of Sociology and Social Policy.

